# Correction to ‘Heat Shock Transcription Factor 3 Regulates Plant Immune Response Through Modulation of Salicylic Acid Accumulation and Signalling in Cassava’

**DOI:** 10.1111/mpp.70032

**Published:** 2024-12-09

## Abstract

Wei, Y.X., Liu, G.Y., Chang, Y.L., He, C.Z., Shi, H.T. Heat Shock Transcription Factor 3 Regulates Plant Immune Response Through Modulation of Salicylic Acid Accumulation and Signalling in Cassava. *Molecular Plant Pathology*, 2018; 19: 2209–2220.

In the above article, there were unintentional errors in Figure 3 and Figure 6, specifically in the images of 0 dpi at Figure 3B and 6 dpi (pTRV‐*MeEDS1*, pTRV‐*MePR4*) at Figure 6C.

These errors have been corrected in the below images:

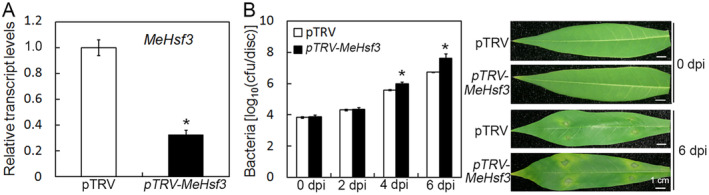

Corrected Figure 3

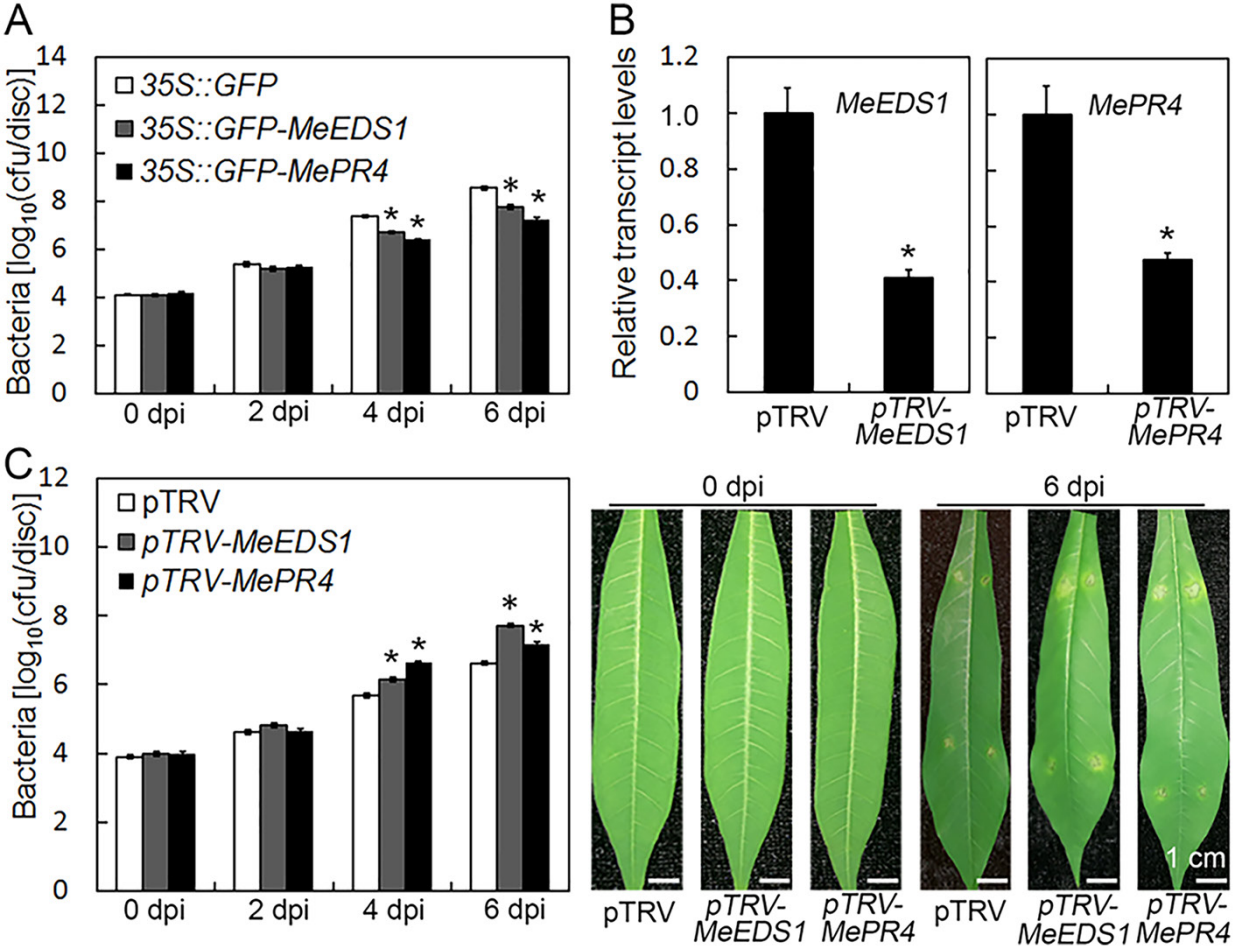

Corrected Figure 6

We apologise for these errors.

Wei, Y.X., Liu, G.Y., Chang, Y.L., He, C.Z., Shi, H.T. Heat Shock Transcription Factor 3 Regulates Plant Immune Response Through Modulation of Salicylic Acid Accumulation and Signalling in Cassava. *Molecular Plant Pathology*, 2018; 19: 2209–2220.

In the above article, there were unintentional errors in Figure 3 and Figure 6, specifically in the images of 0 dpi at Figure 3B and 6 dpi (pTRV‐*MeEDS1*, pTRV‐*MePR4*) at Figure 6C.

These errors have been corrected in the below images:

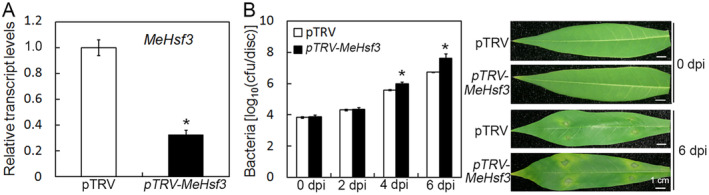

Corrected Figure 3

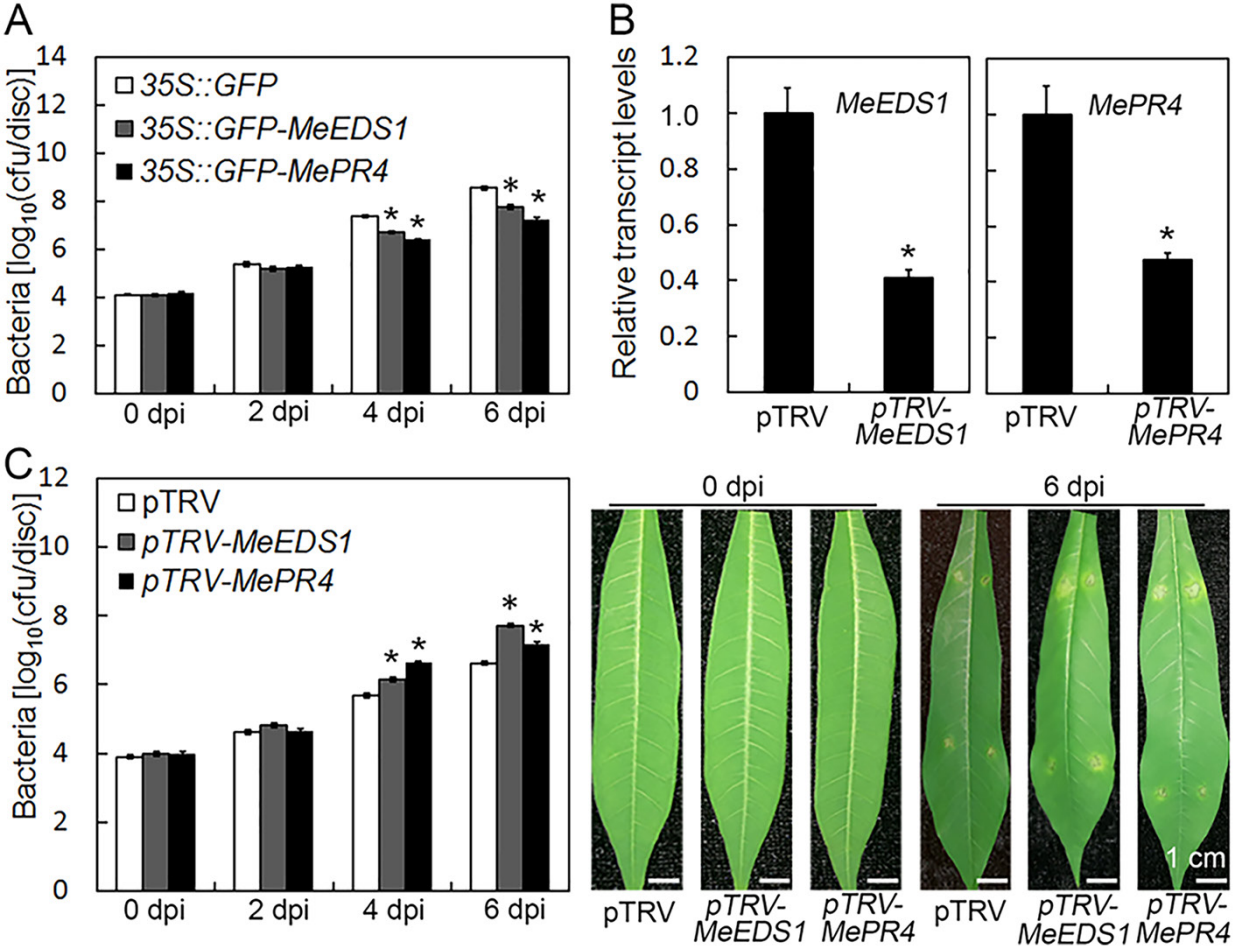

Corrected Figure 6

We apologise for these errors.

